# Inhibitory Effect of Metformin and Pyridoxamine in the Formation of Early, Intermediate and Advanced Glycation End-Products

**DOI:** 10.1371/journal.pone.0072128

**Published:** 2013-09-04

**Authors:** Saheem Ahmad, Uzma Shahab, Mohd. Hassan Baig, Mohd. Sajid Khan, M. Salman Khan, A. K. Srivastava, Mohd Saeed, Moinuddin

**Affiliations:** 1 Department of Biotechnology, Integral University, Lucknow, India; 2 Department of Biochemistry, J.N. Medical College, Faculty of Medicine, Aligarh Muslim University, Aligarh, India; Oak Ridge National Laboratory, United States of America

## Abstract

**Background:**

Non-enzymatic glycation is the addition of free carbonyl group of reducing sugar to the free amino groups of proteins, resulting in the formation of a Schiff base and an Amadori product. Dihydroxyacetone (DHA) is one of the carbonyl species which reacts rapidly with the free amino groups of proteins to form advanced glycation end products (AGEs). The highly reactive dihydroxyacetone phosphate is a derivative of dihydroxyacetone (DHA), and a product of glycolysis, having potential glycating effects to form AGEs. The formation of AGEs results in the generation of free radicals which play an important role in the pathophysiology of aging and diabetic complications. While the formation of DHA-AGEs has been demonstrated previously, no extensive studies have been performed to assess the inhibition of AGE inhibitors at all the three stages of glycation (early, intermediate and late) using metformin (MF) and pyridoxamine (PM) as a novel inhibitor.

**Methodology/Principal Findings:**

In this study we report glycation of human serum albumin (HSA) & its characterization by various spectroscopic techniques. Furthermore, inhibition of glycation products at all the stages of glycation was also studied. Spectroscopic analysis suggests structural perturbations in the HSA as a result of modification which might be due to generation of free radicals and formation of AGEs.

**Conclusion:**

The inhibition in the formation of glycation reaction reveals that Pyridoxamine is a better antiglycating agent than Metformin at all stages of the glycation (early, intermediate and late stages).

## Introduction

Glycation is the non-enzymatic reaction of free reducing sugars with free amino groups of proteins, DNA and lipids. The reaction starts with the formation of highly unstable Schiff base, which are then transformed into early glycation product also known as Amadori product [1]. These intermediate undergoes a series of complex reactions, and generate cross-linked and fluorescent derivatives known as advanced glycation end products (AGEs). AGEs accumulate in vascular wall tissues and on plasma lipoproteins and bind to AGE specific receptors (RAGEs) with ageing. AGEs bind to RAGE at an accelerated rate in diabetic patients and play an important role in the development of diabetes complications, age-related cardiovascular disease and osteoarthritis [2]–[4]. It is well established that methylglyoxal (MG) forms AGEs by reacting with bio-macromolecules such as DNA, proteins and lipoproteins [5]–[7]. A number of studies with human subjects have shown that diet-derived AGEs precursors, such as N-ε-carboxymethyllysine (CML) and MG are found to enhance inflammatory responses and oxidative stress in individuals afflicted with debilitating diseases, such as diabetes [8], [9]. Thus, CML and MG are glycation intermediates and precursors of AGEs, and relevant targets for compounds aimed at reducing the undesirable consequences of protein glycation both *in vitro* and *in vivo*.

There is a considerable body of evidence implicating formation and accumulation of AGEs as a major factor in the development of diabetic complications, atherosclerosis, Alzheimer’s disease, and the normal aging process [9]. The significance of this phenomenon becomes more evident where tight association of lipoxidation reactions, over-production of reactive oxygen species (oxidant stress), and over-generation of reactive carbonyl species (RCS) (carbonyl-stress) with the process of AGE formation are considered [3,4&17]. Furthermore, tissue damage particularly in vascular endothelial cells may originate by triggering of key cell signaling systems and stimulation of inappropriate cellular activities through secretion of cytokines and vascular cell adhesion molecules. Thus, therapeutic interventions should not only target AGE formation and AGE-protein cross-link formation. In view of above concern we have compared the inhibition effect of metformin (MF), pyridoxamine (PM) alongwith a well know inhibitor, aminoguanidine (AG).

The pyridoxamine (PM), which is a vitamin B6 metabolite, has proven to be a potent inhibitor of the formation of AGEs in *in vitro* and animal experiments [10]. This effect of PM is most probably due to blockage of the oxidative degradation of the glucose derived Amadori intermediate or due to quenching of the dicarbonyl compounds [11]. However, the clinical evidence on the potential AGE-inhibiting effects of these B6 vitamers is still limited [12]. On the other hand metformin (MF), is one of the first drugs used for antihyperglycemic effects on type 2 diabetes which also shows interaction with diarbonyl compounds and inhibits AGEs formation [13].

Furthermore, the phosphate form of dihydroxyacetone (DHA), i.e., dihydroxyacetone phosphate (DHAP) takes part in glycolysis, and it is an intermediate product of fructose metabolism [14]. The non-enzymatic reactions of proteins with glucose have been extensively reported to occur mainly via lysine and arginine residues of human serum albumin (HSA) and immunoglobulin-G (IgG) [15], [16]. Moreover, glucose induced DNA glycation is also well reported previously, which is primarily at the guanine residues of the DNA [17]. DHA is also reported to contribute to the rapid formation of AGEs and caused damage to DNA in cultured epidermal keratinocytes [18], [19]. Thus, the implications of DHA adduct formation warrant further study in the context of the severity of glycation at the early, intermediate and advanced stages of the reaction.

In the present study commercially available HSA was incubated with 20 mM DHA for 5–25 days of incubation time period under strictly sterile condition. The structural changes induced in HSA were characterized by various spectroscopic techniques. The potential *in vitro* inhibitory activity of PM and MF against early, mid- and late stage of advanced glycation end products formation was also studied using δ- Glu, HSA-DHA and N-acetyl-glycyl-lysine methyl ester (GK-peptide) ribose assay. Furthermore, lactate dehydrogenase (LDH) assay was also performed to see the cytotoxicity of inhibitors at concentrations used in this study.

## Materials and Methods

### Ethical Statement

5 mL of fresh human blood samples were obtained from each five healthy human volunteers after the informed verbal consent. According to the Indian council for medical research, New Delhi, India, Chapter-II, page no. 11–12, the ethical approval for this research was not deemed to be necessary. According to this guideline, Proposals which present less than minimal risk are exempted from the ethical review process.

### 2.1. Chemicals and Materials

5 mL of blood samples were taken in citrate dextrose preparations. Human serum albumin (HSA), N-acetyl-glycyl-lysine methyl ester (G–K peptide), pyridoxamine, metformin, aminoguanidine, methylglyoxal (MG), ribose, and DHA were obtained from Sigma Chemical Co. (St. Louis, MO). The lactate dehydrogenase (LDH) kit was obtained from Biomedical Research Services (Buffalo, NY). 5,5′-dithio-bis-[2-nitrobenzoic acid] (DTNB) was obtained from Pierce Biotechnology (Rockford, IL). All other reagents were of highest analytical grade available.

### 2.2. Glycation of HSA

Human serum albumin was glycated using dihydroxyacetone (DHA) as a glycating agent. The reaction mixtures contained 20 µM HSA with 20 mM DHA in a final volume of 3 mL of the 20 mM sodium phosphate buffer, pH 7.4 containing 150 mM NaCl and incubated at 37°C for different time intervals (5–25 days).

### 2.3. Spectroscopic Analysis

The ultraviolet absorption profile of native and glycated HSA was performed as described previously [20], [21].

### 2.4. Fluorescence Analysis

Fluorescence emission profile of native and glycated HSA were recorded on Shimadzu RF-5301 spectrofluorometer. Both native and glycated HSA were excited at 370 nm and emission profile was recorded at 435 nm [3].

### 2.5. Circular Dichroism Measurements

Circular dichroism (CD) profile of native and glycated HSA were recorded on spectropolarimeter (Jasco J-815) in a 1 cm path length cell at 25°C. The wavelength range was from 200 nm to 250 nm and all the scans were recorded at an interval of 0.2 nm [22], [23].

### 2.6. Hemoglobin-δ-gluconolactone (δ-Glu) Assay

The Hemoglobin-δ-gluconolactone (δ-Glu) assay was performed as per previously published procedure [24]. All the blood samples were analyzed in triplicates. The percent inhibition of HbA_1C_ formation by the compound was calculated by: (*B*-*C*)/(*B*-*A*)X100, where *A* is HbA_1C_ concentration in the baseline control tube not treated with δ-Glu, *B* is the HbA_1C_ content of the test tube treated with δ-Glu, and *C* is the HbA_1C_ levels in the sample treated with both δ-Glu and the compound under study.

### 2.7. MG-HSA Assay

The method of Lee *et al*., was followed with some minor modifications [25]. Briefly, HSA (50 mg/ml) was incubated with 40 mM MG in the absence or presence of 100 or 200 µM metformin and 10 or 20 mM pyridoxamine under sterile conditions in 100 mM phosphate buffer (pH 7.4) at 37°C for 14 days.

The % inhibition of AGEs formation = [1 −(fluorescence of the test group/fluorescence of the control group)] × 100.

### 2.8. HSA-DHA Assay

The method of Rahbar *et al*., was followed with slight modifications for the determination of percent of AGEs formed [26].

### 2.9. N-Acetyl-glycyl-lysine Methyl Ester (GK peptide)-ribose Assay

GK-peptide-ribose assay was performed with slight modifications from the previously published method [27]. Briefly, GK peptide (50 mg/ml) was incubated with 100 mM ribose in 100 mM sodium phosphate buffer, pH 7.4 containing 0.02% NaN_3_ in the presence or absence of 200 µM MF or 20 mM PM. The fluorescence of the mixture was read at 340 nm excitation wavelength and 420 nm emission wavelengths using a Shimadzu RF-5301 spectrofluorometer.

### 2.10. Lactate Dehydrogenase (LDH) Assay

Platelet rich plasma was obtained from the supernatant resulting from the centrifugation of blood at 200 g at room temperature. Metformin (10–1000 µM) and PM (1–20 mM) were incubated with platelet for 2 h and the cytotoxicity of the MF on platelets was measured by the release of lactate dehydrogenase (LDH) from platelets suspension lysed with 1% Triton X-100 using the commercially available LDH kit (Biomedical Research Services).

### 2.11. Statistical Analysis

The data were analysed by one-way analysis of variance (ANOVA) followed by Duncan’s multiple range test. In all cases, p<0.01 was used to determine significance.

## Results and Discussions

**Figure 1:**
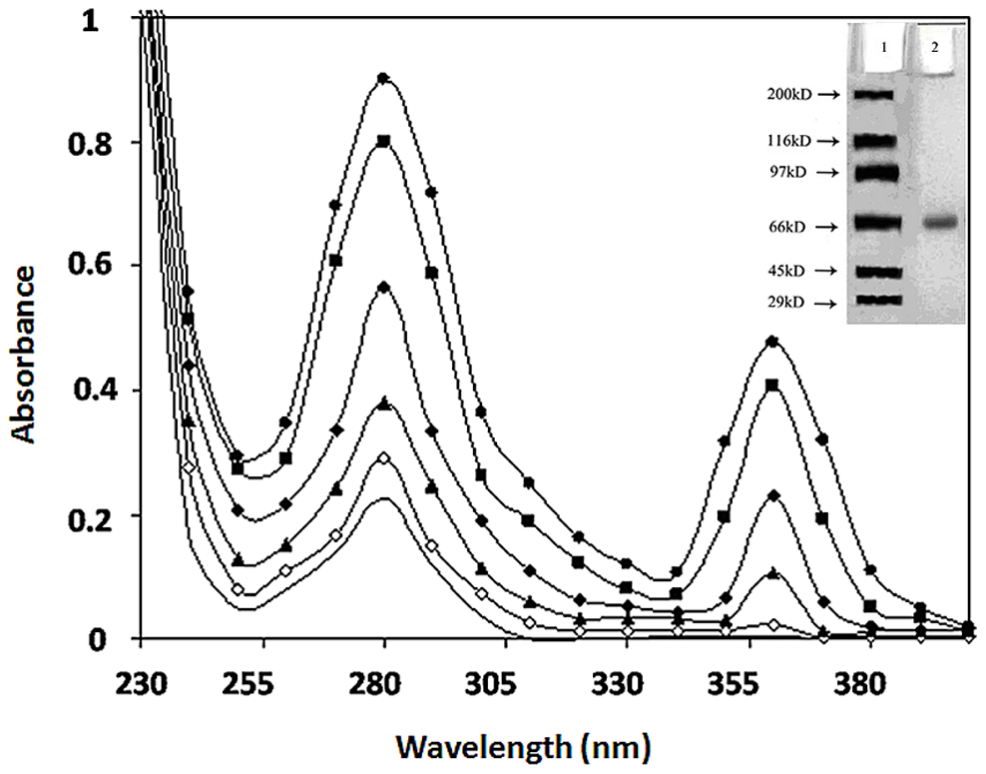
UV-Visible Spectral profile of native HSA (—) and HSA glycated with 20 mM DHA incubated for 5 (-o-), 10 (-▴-), 15(-♦-), 20 (-▪-) and 25 days (-•-).

Pilot experiments were undertaken to work out the time of incubation and optimum concentration of DHA, needed to glycate human serum albumin (HSA). HSA (20 µM) was incubated with and without DHA for different time intervals (5, 10, 15, 20 and 25 days) at 37°C. Native HSA sample when subjected to UV-VIS spectroscopic analysis, a characteristic peak at 280 nm (Fig. 1) and a band of 66 kda on SDS-PAGE was observed (Fig. 1 inset). However, upon modification with DHA; 13, 29, 61, 69 and 71% hyperchromicity was observed at 5, 10, 15, 20 and 25 days of incubation respectively. Maximum hyperchromicity at 280 nm was obtained at 20 mM DHA for 20 days incubation time and further incubation did not result in any significant change in the hyperchromicity (Fig. 1). Therefore, for further characterization, HSA was incubated for 20 days with 20 mM of DHA in phosphate buffer saline.

The observed hyperchromicity could be due to modification of aromatic amino acids or changes in the micro environment of aromatic amino acids. It has been reported that glycation induced AGE-specific absorbance in proteins and their unfolding leading to cross linking and aggregation are responsible for a change in conformation of protein [28]. Furthermore, a new peak was found to appear at 360 nm in the modified HSA samples. This is attributed to the formation of advanced glycation end-products (AGEs) as a result of glycation. Similar extra peak has also been reported upon incubation of HSA with MG (Fig. 1) [29].

**Figure 2:**
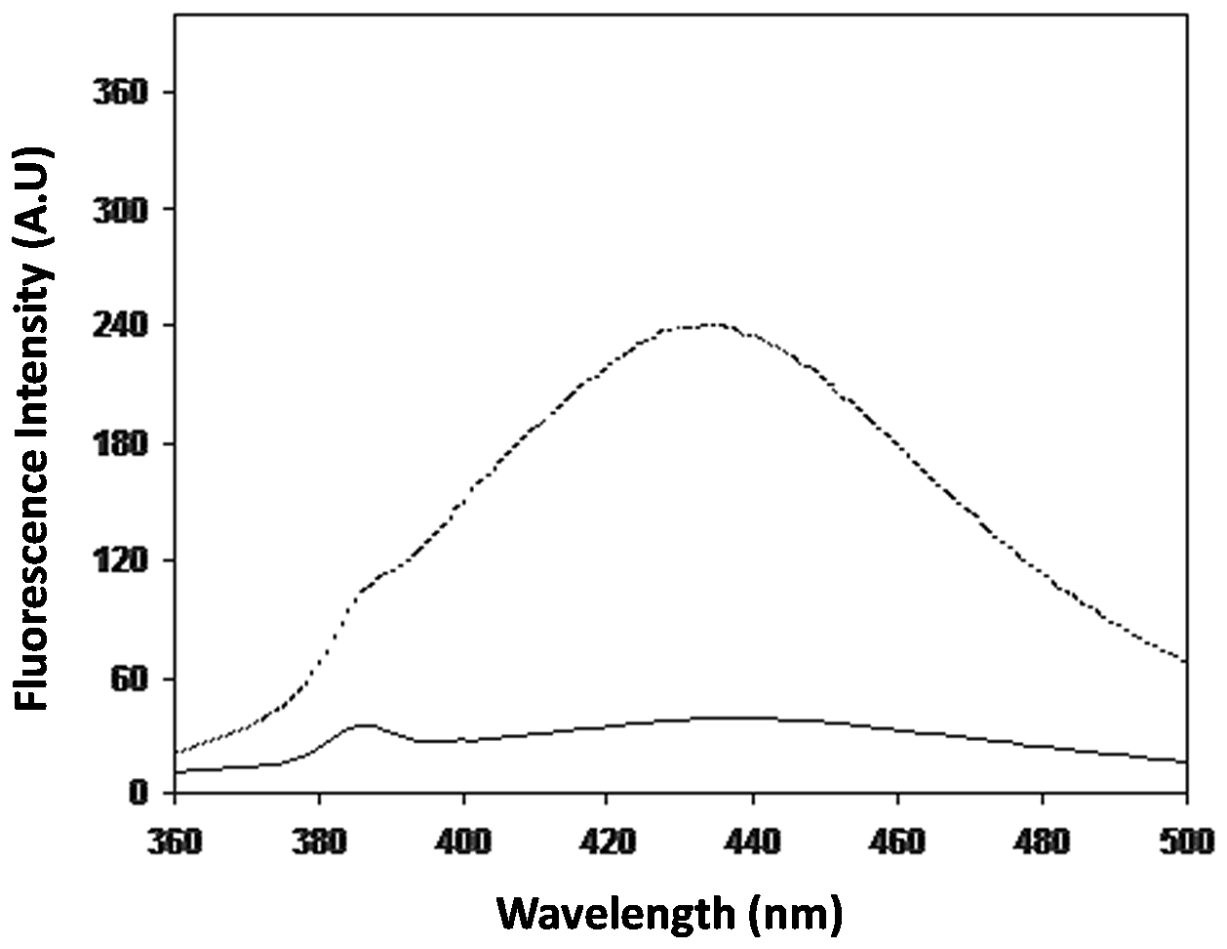
Fluorescence emission spectra of native (—) and AGE- HSA (---). HSA was incubated with 20°C.

Generation of fluorogenic AGEs in glycated-HSA samples were measured using characteristic excitation wavelength of 370 nm. Following excitation, glycated HSA showed emission wavelength of 435 nm. Under identical conditions, native human serum albumin alone does not give any fluorescence. An increase of 83.2% of fluorescence intensity was observed in glycated HSA when compared to its native form (Fig. 2). It suggests that glycation of HSA by DHA generates fluorescent HSA-AGEs, characterized by emission maxima at 435 nm [30].

**Figure 3:**
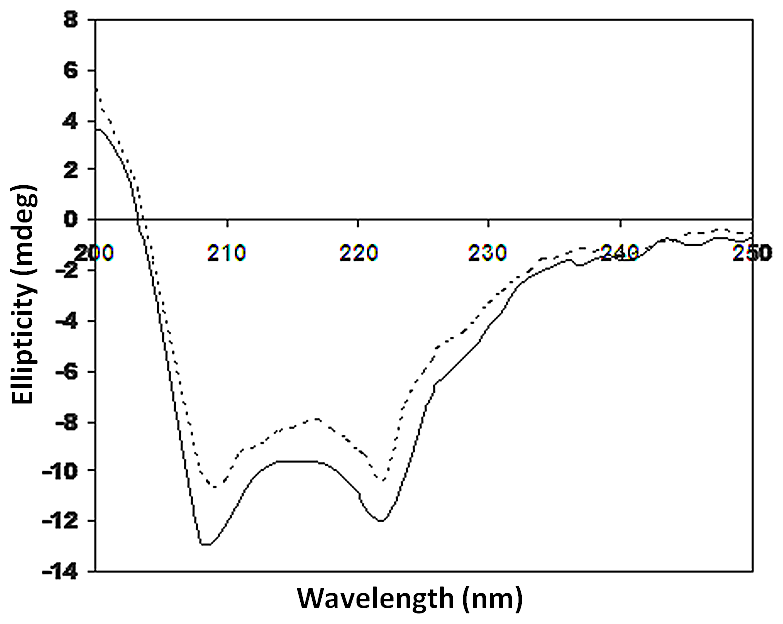
Far UV circular dichroic spectra of native (—) and AGE- HSA (---). HSA was incubated with 20°C.

The Far-UV-CD profile of HSA, was recorded at a wavelength range of 200–250 nm which exhibited a negative peak at 208 nm and 222 nm (Fig. 3). Structural changes in HSA were evaluated by ellipticity measurements. The CD signal of modified analogue shifted from 208 to 210 nm in the direction of higher wavelength, which is indicative of structural changes in HSA pointing towards unwinding of the protein α- helix. When native HSA was compared with the modified one, it showed an increase in ellipticity from −12.76 to −10.00 mdeg at 208 nm and −11.94 to −10.40 mdeg at 222 nm wavelength. This increase in ellipticity corresponds to structural loss in the glycated HSA. The structural loss in HSA after modification might be due to glycation of amino residues (lysine/arginine) of the HSA macromolecule.

**Figure 4:**
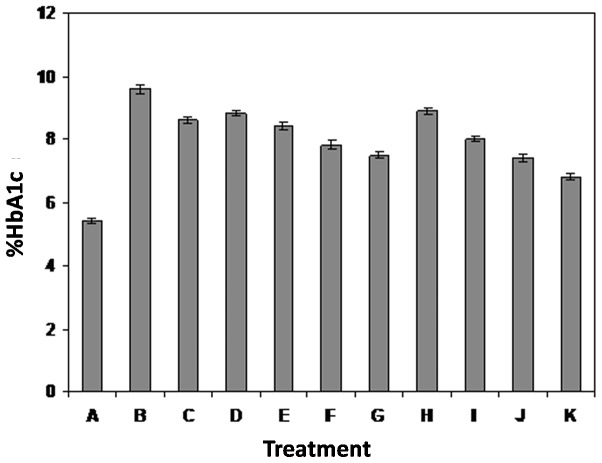
Hemoglobin-δ-gluconolactone assay. Results are mean ±SD and were compared with untreated blood (A = control blood; B = blood+δ-glu; C = blood+δ-glu+10 mM AG; D = blood+δ-glu+10 µM MF; E = blood+δ-glu+100 µM MF; F = blood+δ-glu+500 µM MF; G = blood+ δ-glu+1 mM MF; H = blood+ δ-glu+1 mM PM; I = blood+ δ-glu+5 mM PM; J = blood+ δ-glu+10 mM PM and K = blood+δ-glu+20 mM PM). The various groups are significantly different (p<0.01) from the other.

Hemoglobin-δ-gluconolactone (δ-Glu), an oxidized analogue of glucose, can react rapidly with hemoglobin within the red cells and significantly increases HbA_1C_ levels within hours after incubation. δ-Glu is a specific assay for the investigation of early stage of glycation. This finding was used to measure early stage glycation of hemoglobin (Amadori product) and an assay to evaluate the ability of an inhibitor to inhibit HbA_1C_ formation [31]. δ-glu assay results show that after 20 days of incubation at 37**°**C, there was a marked increase of glycated hemoglobin HbA1c (Fig. 4). At 10 mM, Aminoguanidine (AG) inhibited the formation of AGEs by 15%. Previous published literature [32] indicated that AG had no significant effect of hemoglobin glycation. The mixtures containing hemoglobin-δ-Glu-MF and hemoglobin-δ-Glu-PM showed dose-dependent inhibition of AGE products formation (Fig. 4). At a concentration of 10, 100, 500 and 1000 µM of MF, the inhibition in the formation of HbA1c was 11.6, 17.1, 23.3 and 28.1% respectively. However at 1, 5, 10 and 20 mM concentrations for PM, the inhibition was recorded to be 9.1, 20.4, 29.3 and 41.1% respectively. This result shows that PM inhibited strongly as compared to the MF (Fig. 4, treatment K). Pre-incubation of fresh blood with 20 mM of PM for 24 h followed by addition of δ-gluconolactone resulted in 47% inhibition of the formation of fluorescent products as compared to 41% of inhibition from the simultaneous incubation of whole blood, δ-gluconolactone and 10 mM of PM (data not shown). However, Pre-incubation of fresh blood with 1000 µM of MF for 24 h followed by addition of δ-gluconolactone resulted in 30.3% inhibition of the formation of fluorescent products as compared to 28.1% of inhibition from the simultaneous incubation of whole blood, δ-gluconolactone and 1000 µM of MF. This suggests that pyridoxamine have greater role in the inhibition of the early AGEs formation and the enhanced inhibition of PM is attributed to its increased antioxidant capacity. Since there is the generation of free radical during early stage (Amadori) of glycation [3], [4], therefore PM might have larger impact in the inhibition of glycated analogue of hemoglobin. The clinical relevance of AGE products is that glycated hemoglobin HbA1c isoforms are elevated in diabetic patients [33].

**Figure 5:**
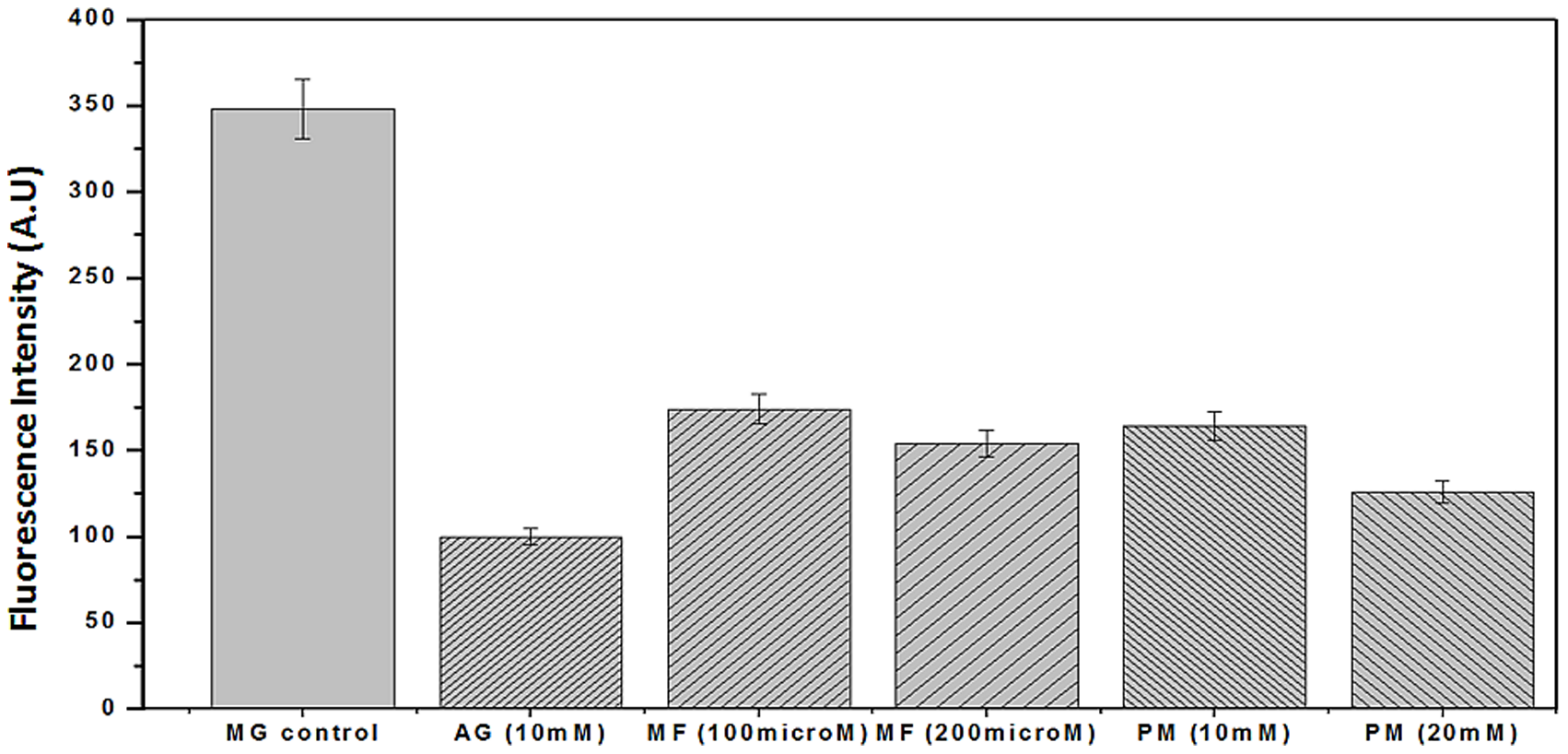
Inhibitory effect of MF and PM on middle (intermediate) stage of HSA glycation by DHA. HSA was incubated with 40& 200 µM MF and 10 & 20 mM PM under sterile conditions in 100 mM phosphate buffer, pH 7.4 at 37°C for 14 days. Results are expressed as mean ±S.D (n = 3), p<0.001.

MG-HSA assay indicate that PM and MF dose-dependently inhibited MG-mediated HSA glycation. Metformin at 100 or 200 µM significantly inhibited 45% and 58% of MG-catalysed HSA glycation, respectively. Whereas, Pyridoxamine showed enhanced inhibition at 10 and 20 mM concentration which was accounted to be 54% and 69% respectively [Fig. 5]. This result point towards that PM inhibits more than that of MF suggesting that PM has larger role in the inhibition of the intermediate stages of the glycation as compared to that of the MF. Aminoguanidine at 10 mM inhibited 49% of MG mediated HSA glycation. This is due to antioxidant capacity with strong scavenging activities against reactive carbonyl species [34].

**Figure 6:**
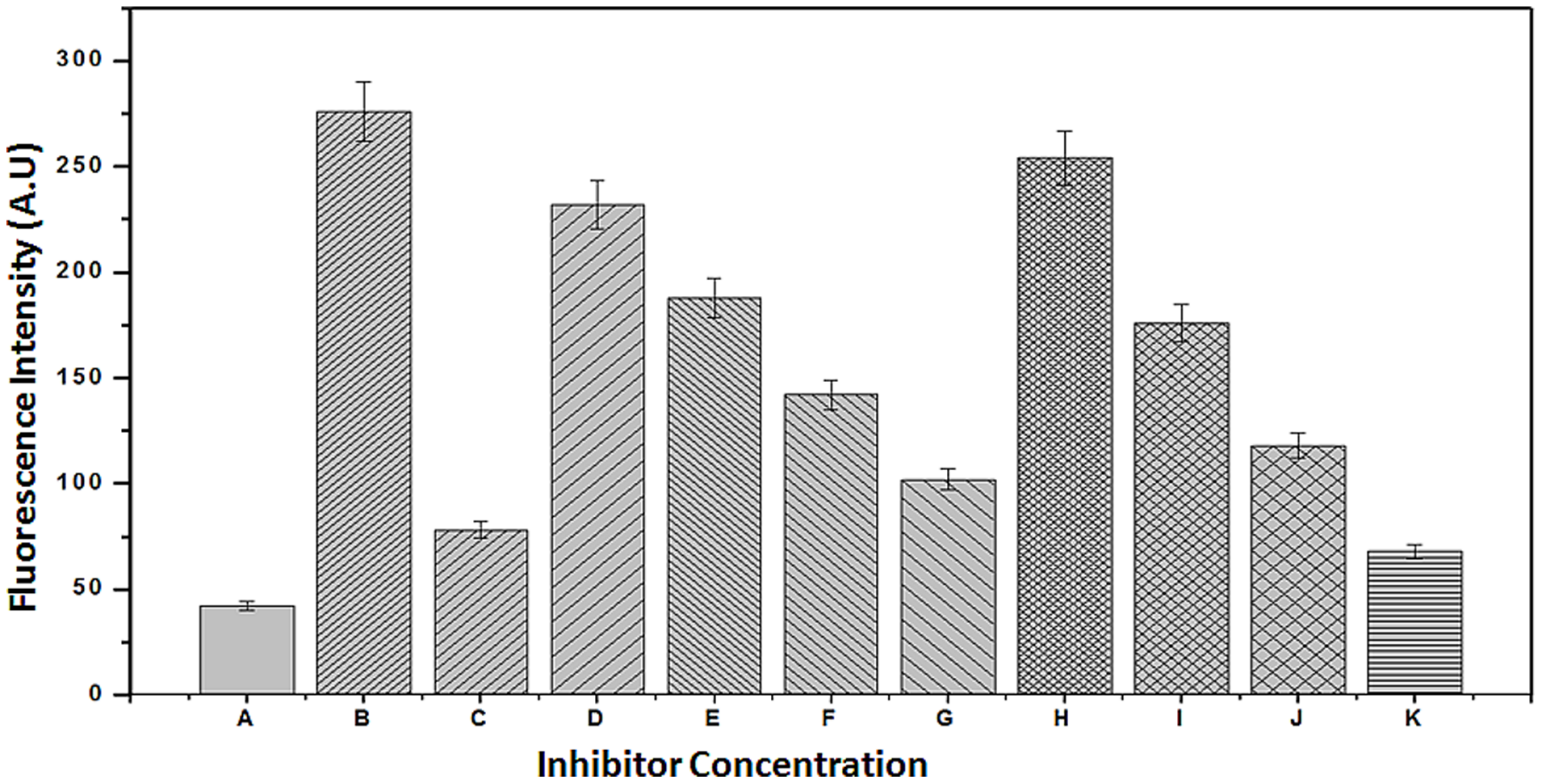
HSA-DHA assay. A = Control HSA; B = HSA+20 mM DHA; C = HSA+20 mM DHA+10 mM AG; D = HSA+20 mM DHA+10 µM MF; E = HSA+20 mM DHA+100 µM MF; F = HSA+20 mM DHA+500 µM MF; G = HSA+20 mM DHA+1000 µM MF; H = HSA+20 mM DHA+1 mM PM; I = HSA+20 mM DHA+5 mM PM; J = HSA+20 mM DHA+10 mM PM and K = HSA+20 mM DHA+20 mM PM. Results are expressed as mean±S.D compared with untreated HSA as a control.

The results of the interactions of HSA and DHA, and HSA–DHA–MF and HSA–DHA–PM are presented in Fig. 6. AGE products are characterized by strong fluorescence intensity at λexc = 370 nm and λem = 440 nm associated with fluorescent compounds, such as pentosidine [35]. At 10 mM concentration, AG inhibited 70.1% of the fluorescence associated with AGE product formation. The results in Fig. 6 also show that MF dose-dependently inhibited the DHA-mediated dependence of fluorescence of HSA. At 1000 µM, MF inhibited almost 73.8% of post-Amadori glycation and AGE product formation. When MF was incubated with HSA before addition of DHA, MF inhibited 80.3% of the post-Amadori glycation and AGE product formation. The inhibitory effect of MF against AGE product formation was stronger than the inhibitory effect of AG at 10 mM. Moreover, PM also showed inhibition (78.4%) in the post-Amadori glycation and AGE formation at a concentration of 20 mM.

**Figure 7:**
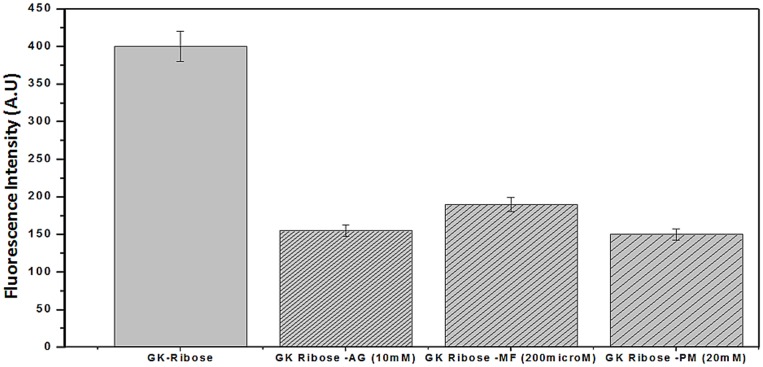
GK-peptide ribose assays. GK- peptide (50 mg/ml) in 100 µL of 500 µM sodium phosphate buffer containing 0.02% NaN_3_ at pH 7.4 was mixed with 0.1 ml of 120 mg/ml ribose in 0.5 M sodium phosphate buffer in the absence or the presence of the 200 µM MF and 20 mM PM and incubated for 14 days at 37°C. The fluorescence of the mixture was read at 340 nm excitation and 430 nm emission wavelength. Results are the mean ±S.D (n = 3), p<0.001.

Evaluation of the advanced glycation products (AGEs), and AGE-inhibition by the new inhibitors was tested by incubation of GK peptide in ribose in the presence or the absence of the agent, followed by determination of chromophores generated in the course of glycation and AGE formation through determination of their specific fluorescence [36]. This test is used to evaluate the ability of the compounds of the present study to inhibit the cross linking of *N*-acetylglycyl-lysine methyl ester in the presence of ribose. The results of the interaction of MF & PM and GK-peptide–ribose are shown in Fig. 7 At 10 mM, AG inhibited 60% of late glycation products. Comparatively, MF at 200 µM inhibited 66% of late glycation end products over a 14 day period. However, PM at 20 mM concentration, showed moderately high inhibition of 79%. The results also suggest that MF and PM both can protect against late advanced glycation end product formation. Amongst these two inhibitors, PM showed enhanced inhibition suggesting it’s most probable role in AGEs inhibition.

Results in Table 1 show that there was no significant increase in LDH released following incubation of platelets with MF and PM. 4.23% of LDH was released in the incubation medium containing hemoglobin, DHA and MF. Metformin induced weak LDH leakage suggesting that 200 µM MF was not cytotoxic. Moreover, pyridoxamine at a concentration of 20 mM released only 3.89% of LDH in the incubation medium. Around 4% LDH leakage is supposed to be weak in cytotoxicity and hence can be said as non-cytotoxic at this concentration [37]. However, above 200 µM concentration of MF the leakage in the LDH was incredibly high, suggesting that the inhibition caused at above threshold concentration (200 µM) is highly cytotoxic. In contrary PM was found to be weak cytotoxic even to the concentration above of 20 mM (Table 2). In order to look into the real toxic concentration of MF and PM, the LDH assay was also performed for MF and PM above the maximum concentration (threshold concnetration) used in this study. Table 2 shows that on increasing the concentration of MF to even 300 µM, the LDH leakage increased to 6.3%, which is supposed to be incredibly high and undesired to use at this high concentration. On contrary PM showed weak toxicity in terms of LDH leakage even up to 30 mM concentration of PM. The LDH leakage at 30 mM was recorded to be 4.45%. Table 2 shows the toxicity assessment of both MF and PM at increasing concentration above the threshold concentration value.

**Table 1:** Effect of metformin (MF), pyridoxamine (PM) and aminoguanidine (AG) on total lactate dehydrogenase released in the incubation medium from HSA-DHA mixtures.

Incubationtime (Min.)	Control	AG (10 mM)	MF (200 µM)	(PM 20 mM)
0	0±0.00	0±0.000	0±0.000	0±0.000
30	0.56±0.312	2.86±0.413	1.23±0.232	1.23±0.235
60	1.25±0.242	4.23±0.432	2.23±0.331	2.11±0.230
90	1.56±0.211	5.81±0.134	3.56±0.237	3.23±0.212
120	2.78±0.234	6.63±0.232	4.23±0.352	3.89±0.421

Results are presented as mean ± SD.

**Table 2:** Effect of metformin (MF) and pyridoxamine (PM) at increasing concentration above threshold value on total lactate dehydrogenase released in the incubation medium from HSA-DHA mixtures.

Inhibitors at increasingConcentration	Incubation timeat 120 min.	
**MF (225 µM)**	4.51±0.312	
**MF (250 µM)**	5.63±0.563	
**MF (300 µM)**	6.37±0.349	
**PM (22 mM)**	3.92±0.683	
**PM (25 mM)**	4.14±0.334	
**PM (30 mM)**	4.45±0.536	

Results are presented as mean ± SD.

Earlier it has been proposed that metformin detoxifies dicarbonyl in a similar way as aminoguanidine, thereby preventing the subsequent production of AGEs [13]. However, in two randomized trials no additional effects of metformin were observed in comparison to other anti-hyperglycemic treatments [38], which suggests that these AGE-inhibiting effects result from an improvement in glycemic control rather than from a specific dicarbonyl detoxification.

Another class of AGE inhibitors, “Amadorin” (the post-Amadori inhibitors), inhibits the conversion of Amadori intermediates to AGE [39]. The first Amadorin identified was pyridoxamine (PM) that showed a great potential for treatment of diabetic nephropathy. It inhibits AGE formation at different levels by scavenging carbonyl products of glucose and lipid degradation, sequestering catalytic metal ions, blocking oxidative degradation of Amadori intermediate, and trapping of ROS. PM has now entered phase III of clinical trial [40]. Furthermore, PM treatment combined with thiamine showed no effect on plasma AGEs and pentosidine [41].Thus, the clinical evidence on the potential AGE-inhibiting effects of these B6 vitamers is still limited.

Furthermore, non-enzymatic glycation is a serious concern for the study as it is directly associated with slow and sweat poison disease; the diabetes. The damage caused to the biological macromolecule is due to both glycation and free radicals generated during the process which have serious implications in several disease states. The preference of PM over MF to inhibit glycation is not only due to its enhanced inhibition activity but also due to its non-cytotoxic nature at 20 mM concentration. It is concluded that PM probably acted by inhibiting the increase in AGE intermediates and indicators of carbonyl stress, such as MG, 3-deoxyglucosone (3 DG) and glyoxal, which were considered the primary source of damage to serum protein, HSA. Overall, the role of antioxidant activity in the mechanism of action of PM is unlikely, but deserves further study. Apart from above inhibitors there is also direct role of antioxidant to inhibit the glycation reaction. It is our hypothesis that the efficacy of these inhibitors can be increased using various bioconjugated inhibitors. Since no work has been done on AGEs inhibition using nanoparticles as drug delivery system, therefore, it would be interesting to see the inhibition of AGEs using bioconjugated inhibitors (PM, MF and Aminoguanidine) with gold nanoparticles. Therefore the also study warrants the use of these inhibitors by bio-conjugating it with gold-nanoparticles. Moreover, recently many published literature points towards to stop this menace “glycation” by using natural plant extracts. This has been hypothesized in our recent publication that some medicinal plant extracts which are having anti-oxidant effect might also have the anti-glycating effect [42], [43].
